# Daxx-Dependent H3.3 Deposition Promotes Double-Strand Breaks Repair by Homologous Recombination

**DOI:** 10.3390/cells15020162

**Published:** 2026-01-16

**Authors:** Laura Zannini, Simona Aliprandi, Domenico Delia, Giacomo Buscemi

**Affiliations:** 1 Istituto di Genetica Molecolare Luigi Luca Cavalli-Sforza, Consiglio Nazionale delle Ricerche (IGM-CNR), Via Abbiategrasso 207, 27100 Pavia, Italy; 2 Dipartimento di Bioscienze, Università degli Studi di Milano, Via Celoria 26, 20133 Milan, Italy; 3 IFOM (Istituto FIRC di Oncologia Molecolare), Via Adamello 16, 20139 Milan, Italy

**Keywords:** ATM, DAXX, DNA repair, double strand breaks, histone variant

## Abstract

DNA double-strand breaks (DSBs) can be induced by cellular byproducts or genotoxic agents. Improper processing of these lesions leads to increased genome instability, which constitutes a hallmark of pathological conditions and fuels carcinogenesis. DSBs are primarily repaired by homologous recombination (HR) and non-homologous end joining (NHEJ) and the proper balance between these two pathways is finely modulated by specific molecular events. Here, we report that the histone chaperone DAXX plays a fundamental role in the response to DSBs. Indeed, in human cells, DSBs induce ATM/ATR-dependent phosphorylation of DAXX on serine 424 and 712 and promote its binding to chromatin and the deposition of the histone variant H3.3 in proximity to DNA breaks. Enrichment of H3.3 at DSBs promotes 53BP1 recruitment to these lesions and the repair of DNA breaks by HR pathways. Moreover, H3.3-specific post translational modifications, particularly K36 tri-methylation, play a key role in these processes. Altogether, these findings indicate that DAXX and H3.3 mutations may contribute to tumorigenesis-enhancing genome instability.

## 1. Introduction

The DNA damage response (DDR) is a complex network of pathways that senses and repairs DNA lesions [1]. DDR alterations are frequent events during tumorigenesis, causing mutation rate increase, hyper-proliferation stress tolerance and prevention of DDR-dependent apoptosis or senescence. DNA double-strand breaks (DSBs) are the most hazardous form of DNA damage due to their ability to trigger chromosomal alterations if they are not repaired in a timely and accurate manner. Since DNA lesions occur in the chromatin landscape, remodelling steps of this structure characterize any stage of the DDR. For this reason, in recent years, several histone post translational modifications (PTMs) and chromatin remodelling factors have been associated with different steps of DNA damage repair.

The case of H3.3 is particularly intriguing. This histone variant represents 10–30% of the total cellular histone H3 pool in human cells and differs from H3.1 for five amino acids and exhibits DNA replication-independent deposition [2]. These differences are sufficient to confer particular properties to H3.3, which are also an effect of peculiar PTMs [3,4]. H3.3 was previously reported to be involved in the response to UV damage [5] and DSBs repair [6]. In the first case, H3.3 was loaded by the histone chaperone HIRA (histone regulator A), and in the second case by an unknown chaperone and the CHD2 remodelling factor.

Apart from HIRA, another histone chaperone, DAXX (or DAP6, death-associated protein six), is responsible for histone variant H3.3 deposition inside chromatin [7]. DAXX is a multifunctional protein that physically interacts with chromatin remodelling enzymes and transcription factors [8]. Initially DAXX was reported to load H3.3 at pericentromeric heterochromatin and telomeres through its interaction with the DNA helicase ATRX [9]. ATRX and DAXX mutations [10] may promote alternative lengthening of telomeres (ALT), which is a homologous recombination-based mechanism that elongates telomeres in telomerase-negative cancer cells. Furthermore, the ATRX/DAXX/H3.3 pathway was found to be involved in the DNA synthesis step of HR [11], thus underlining another role of H3.3 deposition during the DDR. However, DAXX activity is not restricted to its collaboration with ATRX; indeed, ATRX-independent functions that either rely or do not rely on H3.3 have been described for this protein [12,13,14]. Indeed, DAXX participates in the regulation of chromosome structures [12], apoptosis [15], autophagy [16] and protein folding [13]. Considering these functions, it is not surprising that DAXX alterations have been involved in tumorigenesis beyond ALT-positive cancer types.

Here we report for the first time that DAXX, through an early deposition of H3.3 at DSBs, affects the kinetics of 53BP1 recruitment at DSBs and regulates the balance between HR and NHEJ. These events are independent of ATRX activity but are mediated by the ATM/ATR-mediated phosphorylation of conserved serine residues on DAXX and by specific PTMs on H3.3. This novel DAXX function further demonstrates that deregulation of DAXX-dependent H3.3 deposition can lead to low-fidelity repair, thus promoting genome instability.

## 2. Materials and Methods

### 2.1. Cells, Transfections and Treatments

Human osteosarcoma cell line U2OS and human embryonic kidney cell line HEK293T were obtained from the American Type Culture Collection (ATCC, Manassas, VA, USA). EJ5-GFP and DR-GFP U2OS [17] were kind gifts from Prof. J. Stark and Prof. S. Piccolo. These cell lines were all cultured in Dulbecco’s modified Eagle’s medium (DMEM; Lonza, Basel, Switzerland) with 10% fetal bovine serum (FBS). Cells were maintained at 37 °C in a humidified atmosphere containing 5% CO_2_. Stably transduced cell lines were selected in medium containing 600 μg/mL G418, 10 μg/mL blasticidin and 1.8 μg/mL puromycin. To induce DAXX expression, doxocyclin was added at 0.8–1.2 μg/mL. Cells were transfected using Lipofectamine 3000 or RNAiMAX (Thermo Fisher Scientific, Waltham, MA, USA) according to manufacturer instructions. Bleomycin (Sanofi, Paris, France) treatments were performed at 12 or 120 µM. Neocarzinostatin (Sigma-Aldrich, Darmstadt, Germany) was used at a concentration of 0.2 or 0.5 nM, 4-NQO (Sigma-Aldrich) and etoposide (TEVA, Petah Tiqwa, Israel) was added at 10 µM. Cells were irradiated using a ^137^Ce source. The ATM (KU-55933, Sigma-Aldrich) or ATR (VE821, Selleckchem, Houston, TX, USA) inhibitors were added, respectively, at 10 and 2 µM, 1 h before bleomycin.

### 2.2. Antibodies

Antibodies are described and listed in Supplementary Information Table S1.

### 2.3. Expression Vectors and siRNAs

U2OS cell lines silenced for DAXX were obtained by stable transfection of shDAXX sequence cloned in the pENTER U6 vector of the BLOCK-iT™ U6 RNAi Entry Vectors (Thermo Fisher Scientific). Human DAXX cDNA was cloned in the pcDNA3-HA vector for transient transfections or in the pTRE3G vector of the Tet-ON 3G Inducible Expression System for stable transfections. The DAXX cDNA sequence for the Tet-ON system contains two silent mutations within the region targeted by the DAXX shRNA to escape the silencing. Silencing sequences are listed in Supplementary Information Table S2. H3.3-YFP plasmid was obtained from Addgene, Cambridge, MA, USA (#8693). H3.3 gene was successively cloned in the pcDNA3-FLAG vector.

### 2.4. Immunofluorescence and Micronuclei Staining

Immunofluorescence assays were performed as previously described [18]. Antibodies concentrations are listed in Supplementary Information Table S1. EdU staining was performed with the Click-it EdU assay kit (Thermo Fisher Scientific). In situ proximity ligation assays were performed as previously described [19]. Micronuclei were identified by DAPI staining and enumerated as in [20]. Images were captured with a Leica Microsystems DMRA2 microscope equipped with a DFC450C camera. Quantitative immunofluorescence analyses were performed in parallel with identical acquisition parameters among different conditions, using CellProfiler 4.2 software and specific pipelines. In some cases, manual counting was carried out at microscope by two experienced operators who independently generated results from the same samples.

### 2.5. Cell Extracts

Cells were routinely lysed with Laemmli or ELB buffer. Chromatin purification was performed as previously reported [21]. Histones were extracted from chromatin fraction by Laemmli buffer or acid extraction [22]. Western blots were performed with the antibodies listed in Supplementary Information Table S1. Densitometric analyses were performed with the ImageQuant 5.2 software.

### 2.6. Immunoprecipitations

Immunoprecipitations were performed as in [23]. Oligonucleosome preparation and γ-H2AX immunoprecipitation was previously described [24,25].

### 2.7. Chromatin Immunoprecipitation and Quantitative Real-Time PCR

Chromatin immunoprecipitations were performed essentially as described in [26]. Briefly, cells were fixed with 1% formaldehyde, then lysed in RIPA buffer and sonicated with Bioruptor Plus sonication device (Diagenode, Liege, Belgium). Immunoprecipitations were performed with 2 µg of anti-DAXX (Santa Cruz Biotechnology, Dallas, TX, USA) or anti-FLAG-M2 (Sigma-Aldrich) antibodies. Immunoprecipitated DNA was purified with the Chromatin IP DNA Purification kit (Active Motif, Carlsbad, CA, USA) and analyzed by real-time PCR with a 7900 HT Fast Real-Time PCR system (Thermo Fisher Scientific) and Fast SYBR Green Master Mix (Thermo Fisher Scientific). Results were calculated with the percent input method and expressed as the ratio of the DNA immunoprecipitated after SceI expression and the DNA immunoprecipitated from mock transfected cells. Primer used are DR-GFP+500for 5′-AGCTCGCCGACCACTACCAG-3′, DR-GFP+500rev 5′-CGTTGGGGTCTTTGCTCAGG-3′, DR-GFP+1300for 5′-CCCCCGTAGCTCCAATCCTT-3′, DR-GFP+1300rev 5′-CCAGGAGCGGATCGAAATTG-3′, GAPDHfor 5′-AATCCCATCACCATCTTCCA-3′ GAPDHrev 5′-TGGACTCCACGACGTACTCA-3′.

### 2.8. NHEJ and HR Reporter Assays

To evaluate repair pathways incidence, U2OS EJ5-GFP and DR-GFP cells were silenced for DAXX, BRCA1 and control. After 40 h, cells were transfected with the I-SceI expression plasmid (pCBASceI, #26477, Addgene) in combination with a reduced amount of DAXX, BRCA1 or control siRNA; 96 h after the initial silencing, cells were harvested and GFP-positive cells were detected by flow cytometry (more than 20,000 events acquired) using a BD FACSCantoII (Becton-Dickinson, Franklin Lakes, NJ, USA). Data were analyzed using FlowJo v10.

## 3. Results

### 3.1. Histone H3.3 Is Loaded by DAXX at DNA Breaks

**Figure 1:**
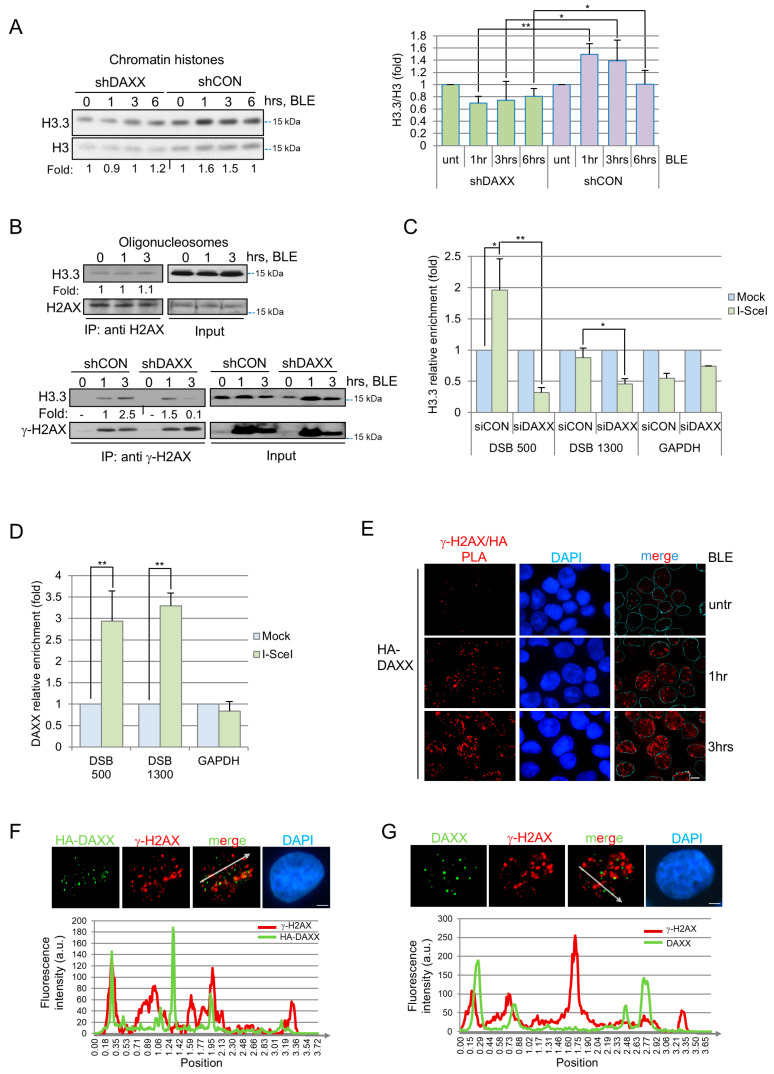
H3.3 histone variant is enriched at DSBs in a DAXX-dependent manner. (**A**) Purified chromatin samples from mock or shDAXX U2OS cells collected at the indicated times after BLE addition were assayed by immunoblot (**left**). H3.3 signals were normalized against H3 and the relative quantification of band intensities is shown as fold change, with a value of 1 considered as the untreated sample. The chart (**right**) represents the means and standard deviations of three independent experiments and statistical significances were obtained with Student’s *t*-test. * *p* < 0.05, ** *p* < 0.01. (**B**) Immunoprecipitations were conducted with specific antibodies against H2AX (**upper**) or γ-H2AX (**lower**) on oligonucleosome preparations obtained from mock or shDAXX U2OS cells chronically exposed to BLE and collected at the indicated time points. Immunoprecipitates (IPs) and total cell extracts (input) were analyzed by immunoblot. H3.3 signals were normalized, respectively, against H2AX (**upper**) or γ-H2AX (**lower**) and the relative quantification of band intensities is shown as fold change, with a value of 1 considered as the untreated sample (**upper**) or the mock 1 h sample (**lower**). (**C**) U2OS cells containing an exogenously introduced I-SceI site were transfected with control (siCON) and DAXX (siDAXX) silencing. After 24 h, cells were transfected with either I-SceI expression vector (+I-SceI) or an empty vector (mock) in combination with a FLAG-H3.3 construct. Chromatin for ChIP analysis was prepared 40 h after the second transfection and immunoprecipitations were conducted with an anti-FLAG antibody. Quantitative PCRs were performed with primers at 500 or 1300 bp from the break or localized in the GAPDH gene. Real-time PCR values, normalized to input DNAs and to the values obtained with unrelated IgG, were considered as 1 for mock testing. Fold induction for I-SceI samples was calculated and the mean of three independent experiments were plotted. Statistical significances were obtained with Student’s *t*-test. * *p* < 0.05, ** *p* < 0.01. (**D**) U2OS cells containing an exogenously introduced I-SceI site were transfected with a I-SceI expression vector (+I-SceI) or an empty vector (mock). Chromatin for ChIP analysis was prepared 40 h after the transfection and immunoprecipitation was conducted with an anti-DAXX antibody. Quantitative PCR, calculations and plotting were performed as in (**C**), ** *p* < 0.01. (**E**) DAXX interaction with DSBs marker γ-H2AX detected by in situ PLA. Cells expressing HA-DAXX^WT^ were left untreated (untr) or exposed to 12 μM BLE for the indicated time. The interactions were visualized as red fluorescent spots. Nuclei were stained with DAPI (blue). Scale bar: 10 μM. (**F**) U2OS cells or (**G**) U2OS cells overexpressing DAXX^WT^ were treated with 12 μM BLE for 3 h, fixed and tested by immunofluorescence with DAXX (green) and γ-H2AX (red) specific antibodies. Nuclei were stained with DAPI (blue). Scale bar: 2 μM. The graph shows the relative intensities for the green and red channel (a.u. = arbitrary unit) along the line scan (white arrow) to estimate colocalization.

To investigate a possible role for H3.3 at DNA breaks, we initially evaluated the ratio of H3.3 to total H3 in chromatin at different time points after exposure of U2OS (ATRX null, [27]), HEK293T or MRC5 (both ATRX wt) cells to the DSB-inducing agent bleomycin (BLE). In these cells, we observed that H3.3 accumulates at 3 h after drug exposure and this increase is already detectable at 1 hr in U2OS and HEK293T cells (Figure 1A shCON samples and Supplementary Figure S1A). Therefore, H3.3 accumulation in chromatin after DNA damage occurs both in ATRX null and wt cells, thus excluding the role of ATRX in this specific event.

Since DAXX and HIRA can promote replication-independent H3.3 loading [2], we investigated their role in DNA damage-induced H3.3 incorporation. After silencing DAXX in U2OS (Supplementary Figure S1B) we noted that upon BLE treatment, H3.3 loading on damaged chromatin is strongly reduced (Figure 1A), while this is not true for HIRA knock-down (Supplementary Figure S1C,D). Moreover, the impact of DAXX depletion on H3.3 loading was not a consequence of an overall perturbation of the global amount of H3.3 present in the cells (Supplementary Figure S1E). Collectively these data suggest that, at early time points after DNA breaks induction, DAXX promotes chromatin accumulation of histone H3.3 in an ATRX-independent manner.

To determine if H3.3 deposition upon DSBs is widespread or localized at damage sites, we evaluated the presence of H3.3 in purified oligonucleosomes [24] containing H2AX or γ-H2AX; the latter is the phosphorylated H2AX counterpart enriched at DNA breaks [28]. We found that H3.3-H2AX association is not affected by BLE treatment (Figure 1B top), while the fraction of H3.3 co-purifying with γ-H2AX rises between 1 and 3 h after treatment (Figure 1B, lower panels, shCON samples), suggesting an increased deposition of this histone nearby DSBs. Notably, γ-H2AX-H3.3 association strongly decreased between 1 and 3 h in shDAXX cells that received similar treatment (Figure 1B, lower panels, shDAXX samples).

To confirm these findings, we performed chromatin immunoprecipitations (ChIPs) in U2OS cells expressing FLAG-H3.3 and containing an I-SceI site that, once cut by I-SceI endonuclease, generates a DSB [17]. Quantitative real-time PCR with primers located at 500 and 1300 nucleotides from the break, revealed that H3.3 accumulates mainly at 500 bp from the break, and, not at GAPDH gene (Figure 1C), which is consistent with previous observations [29]. Notably, in siDAXX cells, H3.3 enrichment is undetectable around 500 and strongly reduced at 1300 nucleotides from the break (Figure 1C), suggesting that DAXX activity is important for H3.3 accumulation and maintenance nearby the damage.

To determine if DAXX also localizes at DNA lesions, we performed ChIP analyses in U2OS cells bearing the I-SceI cut site, and we observed DAXX accumulation at least up to 1300 nucleotides from the I-SceI break (Figure 1D) and not at the GAPDH gene. To confirm DAXX localization at DNA breaks, we decided to perform in situ Proximity Ligation Assay (PLA) in cells expressing HA-DAXX using anti-HA and γ-H2AX antibodies. Preliminary immunofluorescence analyses revealed that exogenous DAXX was detectable in the nucleus and within PML nuclear bodies, as the endogenous protein (compare Supplementary Figure S1F and Supplementary Figure S1B). After the assays, the presence of PLA-positive dots increasing in number with BLE treatment demonstrates that DAXX and γ-H2AX proteins are in proximity after genotoxic treatment (Figure 1E). It is known that PML bodies are recruited at persisting DNA breaks at later time points after genotoxic treatments [30]. However, DAXX-γ-H2AX PLA signals could not be ascribed exclusively to PML bodies recruitment to damage site, since phosphorylated p53, a protein included in these structures [31], was not found colocalizing with γ-H2AX after BLE treatment by PLA. In addition, immunofluorescence analyses further confirmed DAXX accumulation inside spots that, in a small fraction, are overlapping or juxtaposed with a fraction of γ-H2AX foci in both exogenous (Figure 1F) or endogenous (Figure 1G) conditions.

Collectively, these results suggest that, after genotoxic stress, DAXX is localized at DNA breaks and promotes H3.3 loading at damaged sites.

### 3.2. H3.3 Deposition at Damage Sites Influences 53BP1 Relocalization on DNA Lesions

**Figure 2:**
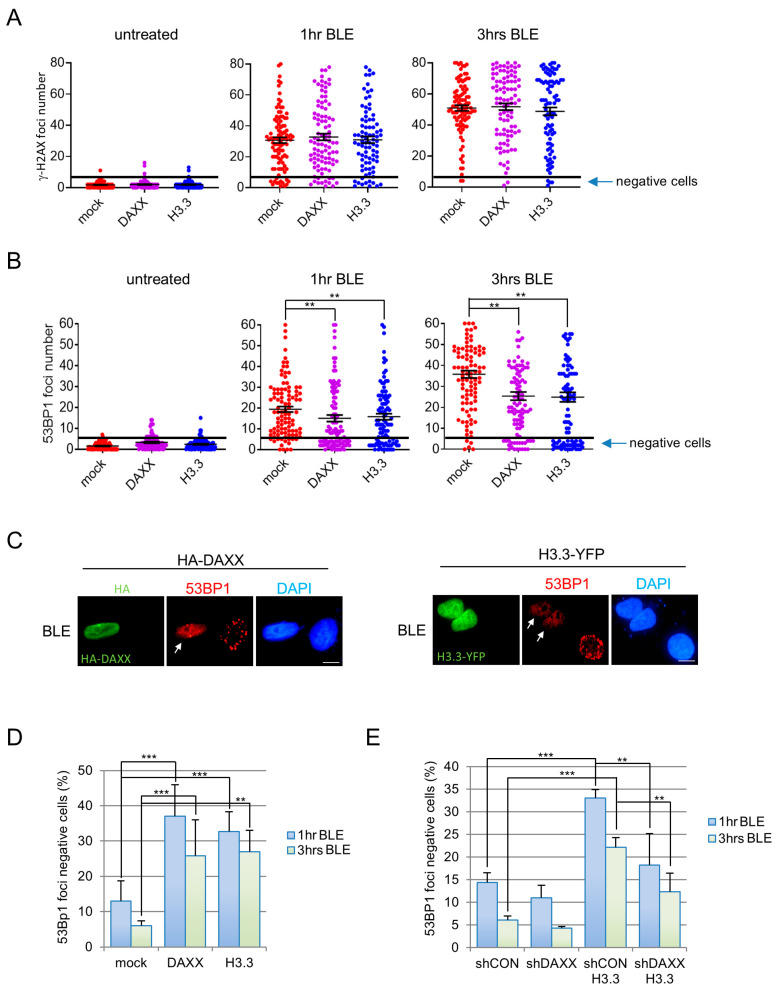
DAXX and H3.3 overexpression impairs 53BP1 foci formation at damage site. (**A**) U2OS cells were transfected with empty vector (mock), HA-DAXX (DAXX) or H3.3-YFP (H3.3). 48 h after transfection cells were treated with 12 μM BLE for the indicated time points, they were successively fixed and immunostained with specific antibodies to enumerate γ-H2AX foci number in cells expressing HA-DAXX or H3.3-YFP. (**B**) Same as (**A**) but for 53BP1 foci. Cells with less than 5 foci (>98% cells in untreated sample) were indicated as negative. (**C**) Examples of cells overexpressing DAXX or H3.3, negative (<5 foci) for 53BP1 foci formation 1 h after 12 μM BLE addition (white arrow). Cells were immunostained for 53BP1 (specific antibody, red) and HA-DAXX (anti-HA antibody, green) or directly analyzed for H3.3-YFP (green) presence. DAPI was used to reveal DNA. Scale bar: 10 μM. (**D**) U2OS cells were transfected with empty vector (mock), HA-DAXX (DAXX) or H3.3-YFP (H3.3) and after 48 h treated with 12 μM BLE for the indicated time points and tested for 53BP1. Cells with less than 5 foci were considered as negative. The chart represents the means and standard deviations (s.d.) of at least three independent experiments. For a single experiment, 300 cells were scored for each cell line. ** *p* < 0.001, *** *p* < 0.0001. (**E**) Same as (**D**) but transfecting shCON or shDAXX U2OS cells with H3.3-YFP and enumerating 53BP1 negative cells at the indicated time points after BLE exposure.

Since chromatin remodelling is a critical early event during the DDR [32], we decided to evaluate if and how DAXX affects the apical steps of this signalling cascade. We started our analyses 1 hr after BLE treatment, which is a time point where parental cells show a significant accumulation of apical events reflective of DSBs formation, like γ-H2AX and 53BP1 foci formation (Supplementary Figure S2A). We initially evaluated the effect of DAXX or H3.3 overexpression on the number of γ-H2AX foci (Supplementary Figure S2B) and no significant changes were detectable in all the tested conditions (Figure 2A). Furthermore, at both 1 and 3 h after treatment we found a limited number of cells that remain negative for γ-H2AX foci (<5 foci as in 95% cells in mock untreated sample) after BLE exposure, and that is not significantly changed by DAXX or H3.3 overexpression (Supplementary Figure S2C).

In the same conditions we analyzed 53BP1 foci formation (Supplementary Figure S2D) which, after DNA damage, is a rapid event promoted by a complex network of histone post translational modifications [33]. We observed that untreated U2OS cells have an average of 1.5 ± 1.4 53BP1 foci and this number increases to 18.9 ± 13.7 and 35.4 ± 18.6, respectively, 1 and 3 h after BLE treatment (Figure 2B mock samples). Interestingly, a significant defect in 53BP1 foci formation was evident following H3.3, or DAXX overexpression (Figure 2B and Supplementary Figure S2E). Looking at the distribution of 53BP1 foci number per cell, we noted that the reduced median number was mainly imputable to an increase in cells that remain negative (<5 spots, as in 99% in untreated cells) for 53BP1 foci formation (Figure 2B,C). Indeed, 1 hr after BLE treatment, 53BP1 negative cells were more than doubled in H3.3 and DAXX overexpressing cells compared to mock and parental cells (Figure 2D). Similarly, at 3 h after BLE, even if the number of 53BP1 decreases further in all samples, H3.3 and DAXX overexpressing cells retained 28% 53BP1 negative cells compared to 5% in control cells (Figure 2D). Importantly, FLAG tagged H3.3 and H2B were also tested and results excluded a role for tag and an unspecific effect of histone overexpression (Supplementary Figure S2F). Altogether, these findings suggest that DAXX or H3.3 overexpression induces the formation of 53BP1 foci in response to BLE treatment. Notably, similar defects in 53BP1 foci formation were obtained overexpressing DAXX and H3.3 in HEK293T cells (Supplementary Figure S2G) and in AsiSI–ER-U20S (Supplementary Figure S2H), where treatment with 4-hydroxytamoxifen induced nuclear localization of AsiSI–ER and DSBs formation [28]. These observations thus exclude a cell type or a DNA break source specificity for 53BP1 foci regulation by DAXX and H3.3. Furthermore, cells’ knock-down for DAXX (shDAXX, Supplementary Figure S2I) showed an amount of 53BP1 foci negative cells comparable to control cells (Figure 2E). Importantly, the accumulation of 53BP1-negative cells induced by H3.3 exogenous expression was completely abolished by DAXX stable (Figure 2E) or transient (Supplementary Figure S2J) depletion but not by HIRA silencing (Supplementary Figure S2J). Therefore, these data demonstrate that the DAXX/H3.3 axis regulates 53BP1 recruitment to DSBs.

### 3.3. DAXX Regulates DNA Repair Pathway Choice, Efficiency and Fidelity Through H3.3

**Figure 3:**
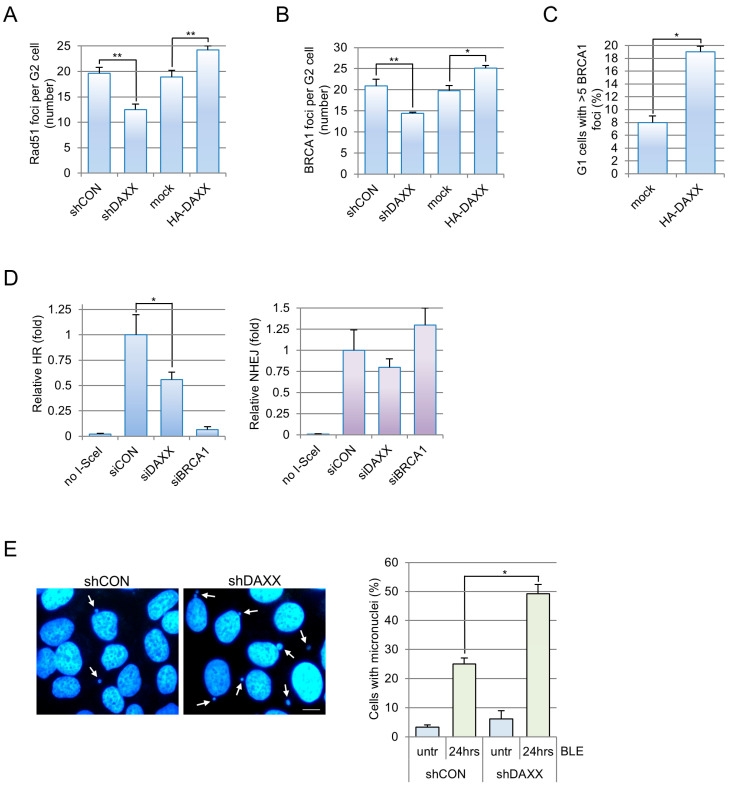
DAXX impacts on DNA repair pathway choice, efficiency and fidelity. (**A**) U2OS cells stably silenced for DAXX (shDAXX) and expressing inducible HA-DAXX WT were immunostained with anti-cyclin B (high cytoplasmic signal reveal G2 cells) and anti-RAD51 antibodies 2 h after the exposure to 12 μM BLE. RAD51 foci were enumerated within cyclin B-positive cells. The graph shows the mean ± s.d. foci number per G2 cells obtained from three independent experiments. At least 100 cells were scored for each experiment. ** *p* < 0.001 statistical significance obtained with Student’s *t*-test. (**B**) Same cells as in (**A**) were immunostained for BRCA1 and cyclin B. The graph shows the mean ± s.d. BRCA1 foci number per G2 cells obtained from three independent experiments. At least 100 cells were scored for each experiment. * *p* < 0.01, ** *p* < 0.001 statistical significance obtained with Student’s *t*-test. (**C**) Mock or HA-DAXX transfected cells were immunostained with anti-cyclin A (nuclear signal reveal S and G2 cells) and anti-BRCA1 antibodies 1 h after the exposure to 12 μM BLE. BRCA1 foci were counted within cells negative for cyclin A (G1 cells). The graph shows the mean percentage ± s.d. of G1 cells with more than 5 foci obtained from three independent experiments. At least 100 G1 cells were scored for each experiment. * *p* < 0.01 statistical significance obtained with Student’s *t*-test. (**D**) DR-GFP-U2OS cells and EJ5-GFP-U2OS cells silenced for DAXX (siDAXX), BRCA1 (siBRCA1, positive control) or control (siCON) were transfected with pCBASceI or empty vector (no I-SceI). After 72 h, samples were analyzed for GFP-positive cells by flow cytometry. The values in the graph are mean ± s.d. of three independent experiments normalized to those of control silenced GFP-positive cells. * *p* < 0.01. (**E**) DAPI staining of DNA was used to reveal micronuclei (**left panel**, white arrows) before and 24 h after neocarzinostatin (NCS) treatment (0.5 nM). Scale bar: 10 μM. The chart (**right panel**) represents the means and standard deviations of at least three independent experiments for control (shCON) or shDAXX cells. For each experiment 1000 cells for sample were scored, * *p* < 0.01.

Since the presence of 53BP1 at DSBs represses HR [34], we speculated that the DAXX-dependent activity on 53BP1 foci formation could influence downstream events in DNA repair, finally unbalancing pathway choice. To test this hypothesis, we focussed our attention on G2 cells where HR and NHEJ, the two main repair pathways for DSBs, are both active [1]. An essential step in HR is the recruitment of RAD51 at the lesion site [35]: we observed, within G2 cells, a significant reduction in RAD51 foci in BLE-treated cells’ knock-down for DAXX and an increase upon DAXX overexpression (Figure 3A and Figure S3A). Similar results were obtained when evaluating BRCA1 foci formation, which is another critical step in HR during the G2 phase [36]. We found that while DAXX depletion leads to a reduction in these foci after damage, the opposite was detectable when we overexpressed DAXX (Figure 3B). It was previously shown that 53BP1 downregulation promotes the occurrence of BRCA1 foci at damage sites in G1 cells [37]. We reasoned that since DAXX overexpression reduces 53BP1 recruitment, it could also share this phenotype. Coherently, we found that DAXX overexpression induces BRCA1 foci formation in 19% of G1 phase cells (Figure 3C). Importantly, all these phenotypes could not be ascribed to the ATRX/DAXX/H3.3 pathway on HR [11], since it occurs in U2OS ATRX null cells. These results suggest that DAXX regulates DNA repair pathway choice and promotes HR through a new mechanism that involves 53BP1 localization. To further confirm these data, we used U2OS cell lines engineered to reveal HR or classic NHEJ repair events [17]. As expected, we found that DAXX silencing, similarly to BRCA1 depletion used as positive control, impaired HR but did not affect classic NHEJ (Figure 3D and Figure S3B). Although these data were obtained in an exponentially growing population they could not be ascribed to alterations of cell cycle progression (Supplementary Figure S3C).

Alterations in DNA repair pathway balancing, particularly regarding the high-fidelity HR pathway, may produce un-ligated or aberrantly repaired DSBs and loss of DNA fragments detectable as micronuclei formation [38]. Therefore, to corroborate our findings about HR impairment in DAXX-depleted cells, we analyzed micronuclei frequency before and after NCS treatment and we found that DAXX absence leads to a significant accumulation of these structures mainly after NCS (Figure 3E). Micronuclei formation is not related to DAXX activity at centromeres since they are mainly negative for the presence of a centromeric marker (Supplementary Figure S3D). Altogether, these data demonstrate an ATRX-independent role for the DAXX/H3.3 axis in HR regulation and fidelity of breaks rejoining.

### 3.4. DAXX Phosphorylation by ATM/ATR Regulates Its Chaperone Activity at DNA Breaks

**Figure 4:**
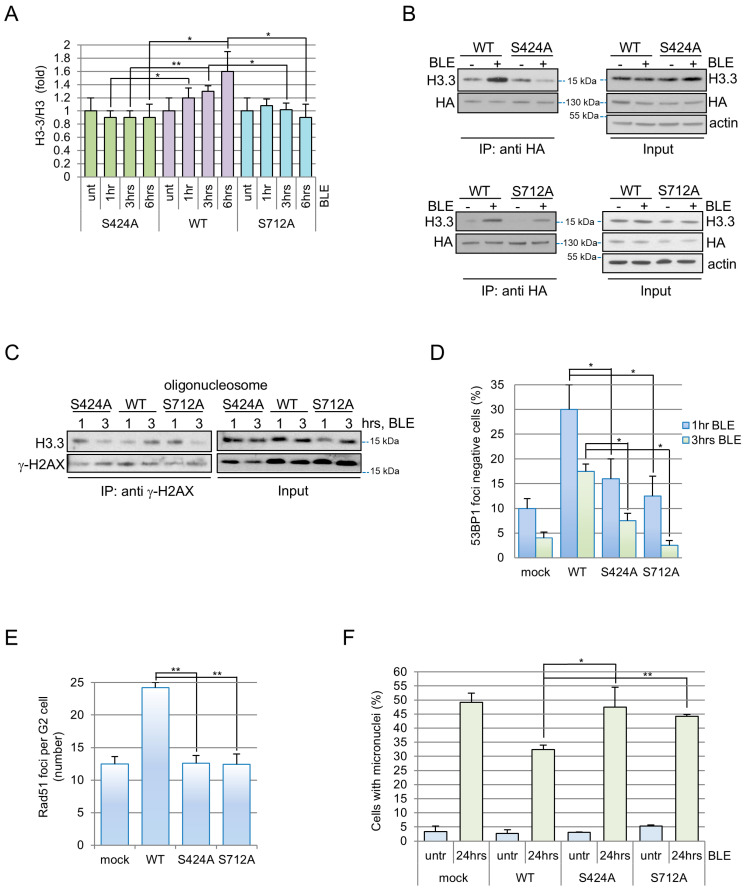
H3.3-DAXX interaction and H3.3 deposition at damage site are impaired by S424 and S712 mutation. (**A**) Purified chromatin samples from U2OS cells, transfected with the indicated forms of DAXX, were assayed by immunoblot. Samples were collected at the indicated times after BLE addition. H3.3 signals were normalized to total H3 levels. Data are shown considering as 1 the untreated sample. The chart represents the means and standard deviations of three independent experiments. (**B**) U2OS cells were transfected with WT or mutant forms of DAXX. Lysates were obtained before (−) and 3 h after BLE exposure (+). HA-DAXX was immunoprecipitated with an anti-HA antibody (IP). H3.3 presence was determined by immunoblot. Actin levels were used as loading control. (**C**) Oligonucleosomes were obtained from U2OS cells expressing a WT or mutant forms of DAXX, exposed for the indicated time to 120 μM BLE. Immunoprecipitation procedures were conducted with a γ-H2AX antibody. Immunocomplexes (IP) and protein levels in total cell extracts (Input) were analyzed by immunoblot. (**D**) U2OS shDAXX+HA-DAXX^WT^, HA-DAXX^S424A^ or HA-DAXX^S712A^ cells fixed 1 and 3 h after 12 μM BLE addition were co-immunostained with anti-HA and -53BP1 antibodies. Cells positive for HA and with less than five 53BP1 foci were considered as negative and results were reported in the chart. At least three independent experiments were conducted and for each experiment 300 cells for sample were scored. (**E**) The same cells as in (**D**) were immunostained with anti-cyclin B (high cytoplasmic signal reveal G2 cells) and anti-RAD51 antibodies 2 h after the exposure to 12 μM BLE. RAD51 foci were enumerated within cyclin B positive cells (see also Figure 3A). The graph shows the mean RAD51 foci number per G2 cells obtained from independent experiments. At least 100 cells were scored for each experiment. (**F**) The same cells as in (**D**) were stained with DAPI to reveal micronuclei before and 24 h after NCS treatment (see also Figure 3E). For (**D**–**F**) graphs the means and s.d. of at least three independent experiments are shown. Statistical significance was obtained with Student’s *t*-test * *p* < 0.01, ** *p* < 0.001.

To investigate the mechanisms regulating DAXX activity towards histone H3.3, we exploited two previously reported independent screenings [39,40] suggesting that ATM/ATR phosphorylate DAXX on the conserved Serine 424 and Serine 712 (S424 and S712) residues upon DNA damage (Supplementary Figure S4A). Initially we produced and validate phosphospecific antibodies confirming that these two residues are effectively phosphorylated after BLE treatment (Supplementary Figure S4B) by ATM/ATR (Supplementary Figure S4C) in human cell lines. Successively, we expressed S424 and S712 Serine to Alanine mutants and we tested DAXX localization by PLA with γ-H2AX after DNA damage induction. We found that DAXX phosphomutants were recruited on DNA lesions as the WT protein (Supplementary Figure S4D). Since these phosphorylation events do not regulate DAXX recruitment at damage site, we investigated whether DAXX phosphorylation might regulate its chaperone activity. To achieve this aim, we generated U2OS cells stably silenced for DAXX (shDAXX) and expressing inducible DAXX phosphomutants resistant to silencing (Supplementary Figure S4E) and we found that cells expressing DAXX^S424A^ or DAXX^S712A^ showed reduced H3.3 presence in chromatin after DNA damage (Figure 4A and Supplementary Figure S4F). Since this phenotype could depend on DAXX association with H3.3, we tested this interaction in cells exposed or not to BLE. We found that DNA breaks strongly promote DAXX^WT^-H3.3 interaction, whereas the induction was absent with DAXX^S424A^ and moderate with DAXX^S712A^ (Figure 4B).

Moreover, co-immunoprecipitation analyses demonstrated that γ-H2AX-H3.3 association rises from 1 to 3 h upon BLE treatment in DAXX^WT^ expressing cells (Figure 4C), while both mutants exhibit a reduction of this association in this time frame (Figure 4C), which is comparable with those observed in shDAXX cells (Figure 1B). Altogether, these data demonstrate that DAXX phosphorylation on S424 and S712 specifically regulates the interaction between DAXX and histone H3.3 and modulates H3.3 loading at damage sites.

As shown above, the exogenous expression of WT DAXX represses 53BP1 foci formation after damage increasing the number of 53BP1-negative cells (Figure 2B–D). Conversely, the expression of DAXX-S424A or -S712A mutants did not significantly increase the number of 53BP1-negative cells at different time points after DNA damage induction (Figure 4D). Coherently, S424A and S712A expression does not alter RAD51 foci formation in the same conditions (Figure 4E) and, finally, contrary to WT expressing cells, the mutant forms of DAXX enhance micronuclei formation after genotoxic treatment (Figure 4F). Collectively these data demonstrate that the ATM/ATR-dependent phosphorylation of DAXX on S424 and S712 regulates DAXX/H3.3 chaperone activity during DDR, thus influencing 53BP1 and RAD51 foci formation, repair pathway choice and fidelity.

### 3.5. H3.3 Methylation at K36 Is Relevant for DAXX- and H3.3-Dependent Regulation of 53BP1 Localization

**Figure 5:**
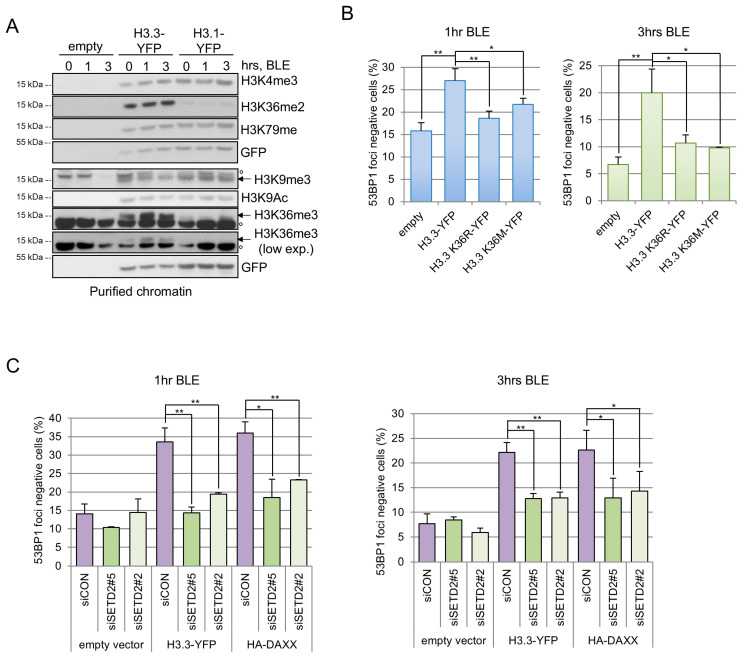
H3.3-K36 methylation contributes to the impairment of 53BP1 recruitment at damage site. (**A**) Post translational modifications of histone H3 were assessed on chromatin extracted from U2OS cells expressing H3.3-YFP, H3.1-YFP or no exogenous histones (empty). Cells were treated for the indicated times with 120 μM BLE. Chromatin was purified from total protein extract and analyzed by immunoblotting. Anti GFP antibody was used to reveal the expression levels of H3.3-YFP and H3.1-YFP. °nonspecific band. (**B**) Cells expressing WT or a mutated form of H3.3-YFP (lysine 36 to arginine or to methionine) were treated with 12 μM BLE and fixed 1 (**left**) or 3 h (**right**) after. 53BP1 foci were enumerated and cells with less than 5 spots considered as negative. At least three independent experiments were conducted and for each experiment 300 cells for sample were scored. Statistical significance was obtained with Student’s *t*-test * *p* < 0.01, ** *p* < 0.001. (**C**) Cells transfected with siCON or siSETD2 silencing in combination with H3.3-YFP or HA-DAXX were tested for 53BP1 foci formation by immunofluorescence after the addition of 12 M BLE. YFP or anti-HA-positive cells were scored for 53BP1 foci and those with less than 5 foci were considered as negative. For a single experiment 300 cells were scored for each cell line. Graph collects the means and s.d. of three independent experiments.

53BP1 recruitment and DNA repair pathway choice are regulated by histone PTMs [33] and since it was previously shown that histone H3.3 has peculiar PTMs, we analyzed H3 modifications in U2OS (Figure 5A) and HEK293T (Supplementary Figure S5A) chromatin. In U2OS cells we found that while K79me, K4me3, K9Ac and K9me3 were present at the same level in H3.3 and H3.1, K36 di- and tri-methylation were enriched in H3.3 histone, (Figure 5A). Similar results were obtained in HEK293T cells testing K36 methylation (Supplementary Figure S5A).

To evaluate the role of K36 PTMs in 53BP1 foci formation, we expressed in U2OS cells the H3.3-K36R mutant, that cannot be methylated, and the H3.3-K36M mutant, a mutation found in more than 90% of human chondroblastoma, which is a benign neoplasia characterized by mutations in the H3.3 gene [41]. Remarkably, while WT H3.3 expression represses 53BP1 spots formation, both K36 mutants show only a limited effect (Figure 5B), suggesting that H3.3K36 PTMs are relevant for 53BP1 recruitment at DNA lesions.

To further test whether H3.3-K36me mediates DAXX activities at damage sites, we silenced SETD2, the histone methyltransferase mainly responsible for K36 tri-methylation [42], thus removing K36me3 on both endogenous and exogenous H3.3 (Supplementary Figure S5B). Intriguingly, we found that reduction in H3.3K36 tri-methylation partially rescued the delayed recruitment of 53BP1 at damage sites caused by DAXX and H3.3 overexpression (Figure 5C). These data therefore indicate a relevant role for K36me3 on DAXX/H3.3 dependent recruitment of 53BP1 at DNA breaks.

## 4. Discussion

Here, we demonstrate that following DSBs formation, histone H3.3 is rapidly loaded by DAXX at damage sites. Such chromatin modification and the associated tri-methylation of H3.3 at lysine 36 modulate 53BP1 recruitment and influence DNA repair pathway choice. This novel function of DAXX is ATRX- and HIRA-independent, but controlled by the apical kinases of the DDR, ATM and ATR, which, after DNA damage, phosphorylate DAXX on at least two new target sites (S424 and S712).

We also found that DAXX itself is recruited to a fraction of DSBs and its depletion, without altering the H3.3 pool, afflicts H3.3 presence for up to 2.5 Kb from break site (considering primers positioning and genomic DNA fragmentation), therefore indicating DAXX relevance in accumulating and/or maintaining H3.3 close to broken DNA ends. Importantly, DAXX and H3.3 accumulation at DNA breaks were observed in cells negative for ATRX, a chromatin remodeler that, in complex with DAXX, plays a role in H3.3 deposition at specific chromosomal regions (telomeres and centromeres) and during the late steps of DNA repair process [11]. We therefore hypothesize that, earlier during DNA breaks detection and repair, an alternative chromatin remodeler could associate with DAXX and stimulate H3.3 deposition at DNA breaks. Notably, since ATRX is deficient in several tumours, investigations about DDR regulations in ATRX-negative cells are of particular interest.

We also demonstrated that increasing the loading of H3.3 on damaged chromatin, through DAXX or H3.3 overexpression, caused a widespread defect in 53BP1 foci formation, an early DDR event influenced by chromatin structure. Unfortunately, since 53BP1 is rapidly recruited at any DSB, this hampered the possibility to detect the increase of 53BP1 foci caused by DAXX depletion. Nonetheless, we found that DAXX knock-down strongly reduced the effect of H3.3 overexpression on 53BP1 foci confirming the mechanistic link among DAXX, H3.3 and 53BP1 accumulation at damage site. Importantly, this effect was not observed after HIRA silencing, demonstrating that this chaperone is irrelevant for H3.3 loading and activity at DSBs.

In addition, our findings demonstrate that the DAXX-dependent incorporation of H3.3 in the DSB region affects 53BP1 recruitment also in cells damaged by AsiSI restriction enzyme, that produces DNA breaks specifically in euchromatin [43]. These data suggest that the DAXX-H3.3 pathway may be preferentially active at breaks in transcribed regions, and these results well agree with the observation that DAXX protein localizes only on a fraction of bleomycin induced DNA breaks.

The localization of 53BP1 on DNA breaks plays a prominent role in regulating DSB repair pathways’ balance, particularly repressing the access of HR factors, like BRCA1, and DNA resection, thus disfavouring the HR pathway [34,44]. Importantly, 53BP1 and BRCA1 compete for binding in a region localized at 1–2 kbs from the break, exactly where we found a prominent DNA damage-dependent deposition of H3.3 by DAXX. Therefore, we asked whether DAXX may affect DNA repair, and we found that the DAXX-dependent defect in 53BP1 recruitment at DSBs unbalances NHEJ/HR pathway usage, facilitating HR. Using a GFP-based approach, we were also able to reveal a reduction in HR activity in DAXX silenced cells, in the absence of any alterations of cell cycle progression, demonstrating that DAXX silencing has the opposite effect of DAXX overexpression on DNA repair pathway choice. Reasonably, these effects of DAXX silencing may be, as for the overexpression, 53BP1-mediated: indeed, DAXX absence, while leaving unaffected the number of 53BP1 foci, could influence the amount or positioning of this protein at DSBs [34], impairing HR. Of note, DAXX-depleted cells do not appear to compensate for the HR defect with a concomitant increase in classical NHEJ. This is probably associated with the fact that other positive signals are required to start this pathway; on the contrary, less regulated and more error prone repair systems could be activated, like single strand annealing or alternative NHEJ.

Unbalancing of DNA repair pathways can lead to a reduction in repair fidelity. Consistently, DAXX silencing, which reduces the high-fidelity system HR, leads to a severe increase in micronuclei formation in cells exposed to an acute damage. Conversely, cells overexpressing DAXX and H3.3 exhibit, in the same conditions, a slightly increased in micronuclei formation, possibly due to highly error prone alternative-NHEJ pathways [45]. These events, occurring in ATRX-negative cells, indicate that genomic rearrangements originate from deregulated repair pathways and not from a DAXX/ATRX-dependent instability at telomeres or centromeres.

To further explore the molecular mechanism regulating DAXX histone chaperone activity during DDR, we took advantage of previously described high-throughput screening that identified DAXX S424 and S712 as putative phosphorylation sites for ATM and ATR [39,40]. Additionally, DAXX-S564 has been demonstrated to be an ATM target site, with a specific function in DAXX/p53 pathway regulation [46]. Here, we demonstrated that S424 and S712 are preferentially targeted by ATM upon DSBs. Moreover, we found that expression of DAXX^WT^, but not phosphomutants, is able to provide the function required for H3.3-γ-H2AX complex formation after damage. These data further corroborate the evidence that H3.3 turnover and accumulation at DSBs substantially relies on the DNA damage-dependent activity of DAXX. Coherently, we observed that expression of DAXX phosphomutants, differently from DAXX^WT^, was unable to alter 53BP1 accumulation at damage site and to promote micronuclei formation. Overall, these data demonstrate the importance of DAXX phosphorylation and that DAXX impact on DDR derives mainly from an altered deposition of H3.3 occurring after lesions appearance. Data obtained with DAXX point mutants also excluded that the effects previously observed on 53BP1 and DNA repair were artefacts of DAXX overexpression.

Next, we asked how the H3.3 chaperone activity of DAXX was modulated upon DNA damage. One hypothesis was that DAXX relocalization on DNA lesion is enough to target H3.3 loading. However, we found that S424 and S712 mutants did not alter DAXX localization at DNA breaks, while, on the contrary, the DNA damage induced DAXX/H3.3 interaction is strongly impaired. These data depict a model where DAXX is recruited at the sites of damage, but it needs site specific, DNA damage- and ATM-dependent phosphorylations to enhance its interaction with H3.3 and modify the chromatin around the lesion. Since S424A and S712 mutants afflict mainly the fraction of DAXX-H3.3 binding that occurs after DNA damage, they leave unaltered DAXX stability, although in vitro experiments showed that H3.3 contributes to DAXX stabilization [47].

We next wondered how H3.3 deposition occurring near a DNA break could influence DNA repair. This histone variant exhibits peculiar PTMs and this may suggest a possible mechanism. Accordingly, we provide evidence that H3.3 inside chromatin accumulates higher levels of K36 di- and tri-methylation compared to H3.1, coherently with previous works [3,4]. Interestingly, mutating K36 residue to arginine or silencing the K36 trimethylase SETD2, we were able to restore the wild-type kinetics of 53BP1 foci formation after damage, in the presence of DAXX or H3.3 overexpression. These results suggest for the first time that H3.3K36me3 contributes to promote a chromatin context that can, directly or indirectly, affect 53BP1 recruitment at sites of damage. Coherently with our results, K36me2 promotes Nbs1/Mre11 complex recruitment at DSBs [48], which is a critical step to start resection during HR. Furthermore, H3K36me3 and SETD2 were described as relevant to promote HR if DSB occurs within transcribed regions [43,49]. Therefore, we propose that H3.3 deposition could contribute to locally maintain or even enrich K36 methylated histones, favouring HR versus NHEJ for the repair of lesions occurring in a specific transcription context.

Strikingly, K36 methylation seems altered in two H3.3 mutations (G34R and G34V) that are common in cerebral hemispheric pediatric glioblastoma [50] and in K36M mutation found in chondroblastoma. Of note, we found that K36M mutation affects H3.3 activity on 53BP1 recruitment. Consistently, about 15% of pediatric glioblastoma showed SETD2 inactivation [51] and SETD2 mutations affect DNA repair in renal cancer [52]. ATRX (or rarely DAXX) mutations were found in 100% of G34-H3.3 mutant cases [50], indicating that ATRX/ALT axis does not overlap with G34 mutations. Therefore, DAXX and H3.3 alterations could favour tumorigenesis through multiple mechanisms: acquisition of ALT, enhanced oncogenes transcription and increased genomic instability due to imprecise repair. Therefore, we propose that the DAXX/H3.3 pathway could be relevant for diagnosis and therapy of some particularly aggressive forms of cancer.

## Data Availability

The data used and/or analyzed during the current study are available from the corresponding author on reasonable request.

## Supplementary Materials

The following supporting information can be downloaded at https://www.mdpi.com/article/10.3390/cells15020162/s1, Figure S1: DAXX/H3.3 axis is regulated by DSBs induction; Figure S2: DAXX-mediated deposition of H3.3 influences the apical events of the DNA damage response to DSBs; Figure S3: DAXX participates in regulating DSBs repair activities; Figure S4: DAXX is phosphorylated by ATM and ATR after genotoxic treatment; Figure S5: H3.3 peculiar PTMs influence 53BP1 accumulation at DSBs; Table S1: Primary antibodies used for western blot (WB) and immunofluorescence (IF); Table S2: sh and siRNA sequences used with references.
