# Supplementary material for: Daxx-Dependent H3.3 Deposition Promotes Double-Strand Breaks Repair by Homologous Recombination
Source: Cells. 2026 Jan 16;15(2):162. doi: 10.3390/cells15020162 (PMC12839749; doi:10.3390/cells15020162)
Supplement: Supplementary file 1 [file cells-15-00162-s001.zip › cells-4040790-supplementary figures.pdf]

Figure S1

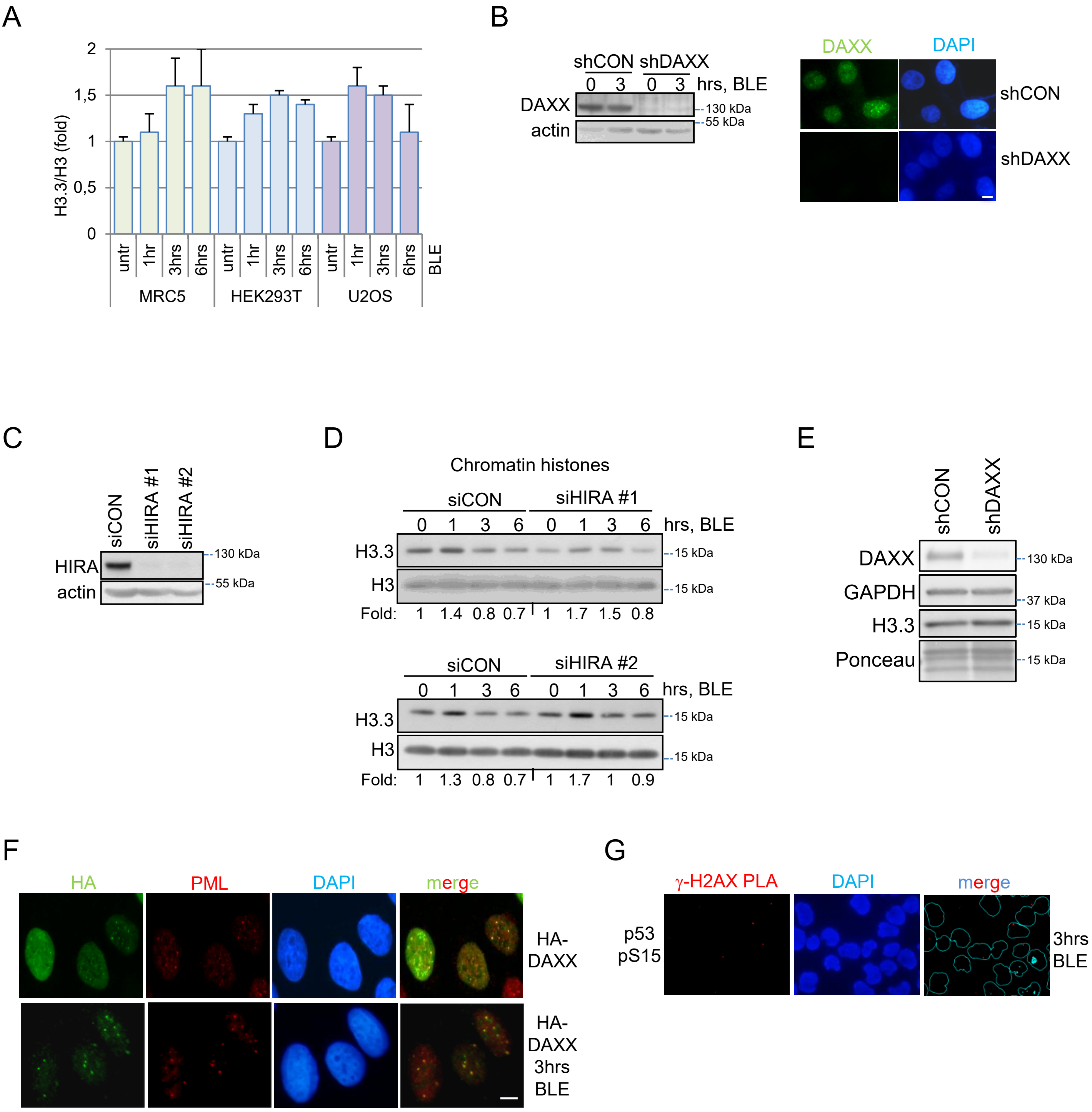

A
H3.3/H3 (fold)
2
1,5
1
0,5
0
untr
1hr
3hrs
6hrs
MRC5
HEK293T
U2OS
BLE
B
shCON shDAXX
0 3 0 3 hrs, BLE
DAXX
actin
130 kDa
55 kDa
DAXX
DAPI
shCON
shDAXX
C
siCON
siHIRA #1
siHIRA #2
HIRA
actin
130 kDa
55 kDa
D
Chromatin histones
siCON
siHIRA #1
0 1 3 6 0 1 3 6 hrs, BLE
H3.3
H3
15 kDa
Fold: 1 1.4 0.8 0.7 1 1.7 1.5 0.8
siCON
siHIRA #2
0 1 3 6 0 1 3 6 hrs, BLE
H3.3
H3
15 kDa
Fold: 1 1.3 0.8 0.7 1 1.7 1 0.9
E
shCON
shDAXX
DAXX
GAPDH
H3.3
Ponceau
130 kDa
37 kDa
15 kDa
F
HA
PML
DAPI
merge
HA-DAXX
HA-DAXX 3hrs BLE
G
γ-H2AX PLA
DAPI
merge
p53 pS15
3hrs BLE

Figure S2

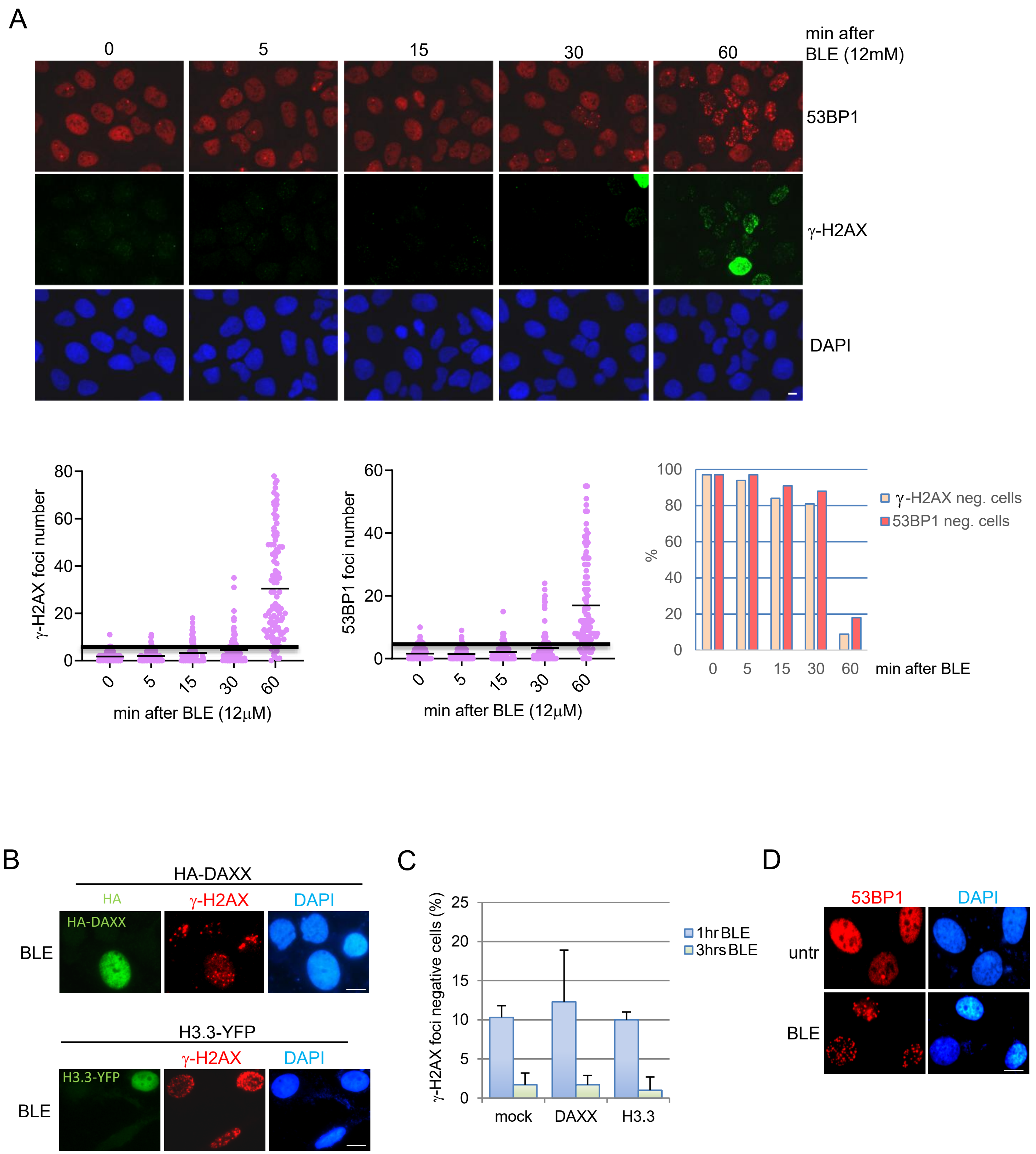
A
min after
BLE (12mM)
0
5
15
30
60
53BP1
γ-H2AX
DAPI
γ-H2AX foci number
min after BLE (12μM)
53BP1 foci number
min after BLE (12μM)
%
γ-H2AX neg. cells
53BP1 neg. cells
min after BLE
B
HA-DAXX
HA
γ-H2AX
DAPI
BLE
HA-DAXX
H3.3-YFP
γ-H2AX
DAPI
BLE
H3.3-YFP
C
γ-H2AX foci negative cells (%)
1hr BLE
3hrs BLE
mock
DAXX
H3.3
D
53BP1
DAPI
untr
BLE

E

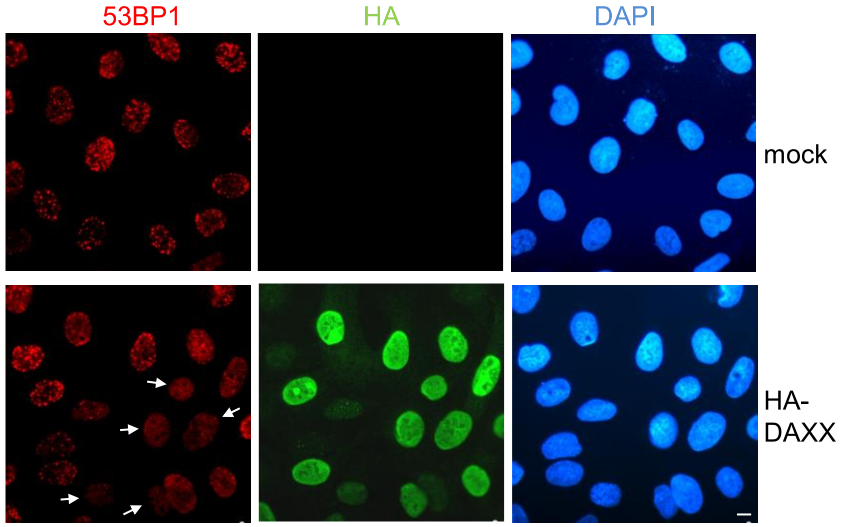
53BP1
HA
DAPI
mock
HA-DAXX

F

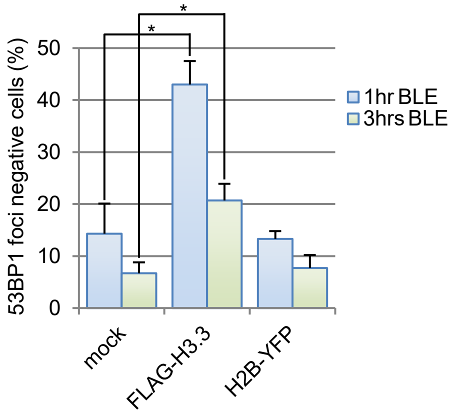
53BP1 foci negative cells (%)
1hr BLE
3hrs BLE
mock
FLAG-H3.3
H2B-YFP
*
*

G

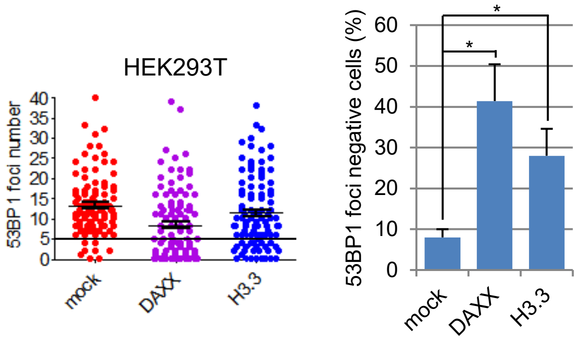
HEK293T
53BP1 foci number
mock
DAXX
H3.3
53BP1 foci negative cells (%)
*
*

H

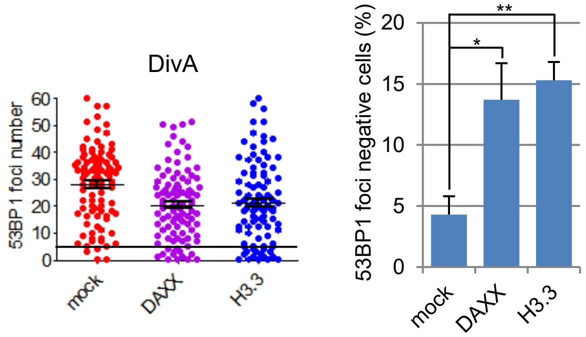
DivA
53BP1 foci number
mock
DAXX
H3.3
53BP1 foci negative cells (%)
*
**

I

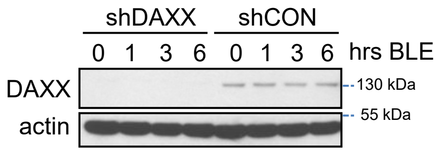
shDAXX
shCON
0 1 3 6 0 1 3 6 hrs BLE
DAXX
actin
130 kDa
55 kDa

J

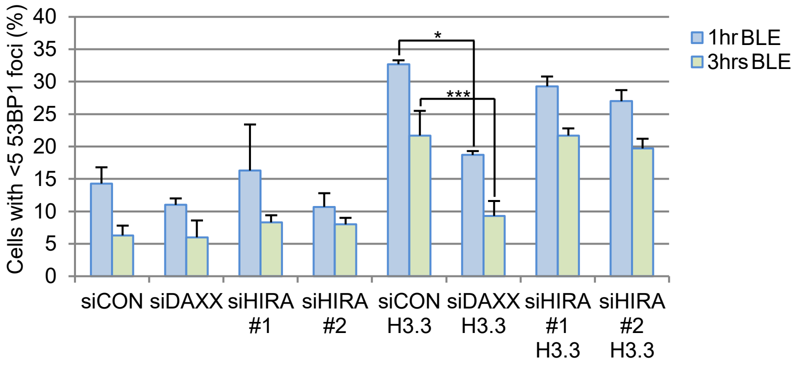
Cells with <5 53BP1 foci (%)
1hr BLE
3hrs BLE
siCON
siDAXX
siHIRA #1
siHIRA #2
siCON H3.3
siDAXX H3.3
siHIRA #1 H3.3
siHIRA #2 H3.3
*
***

Figure S3

A

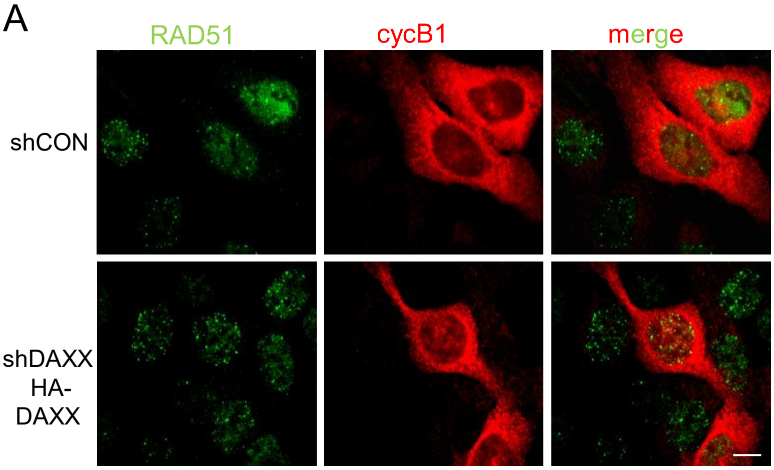

B

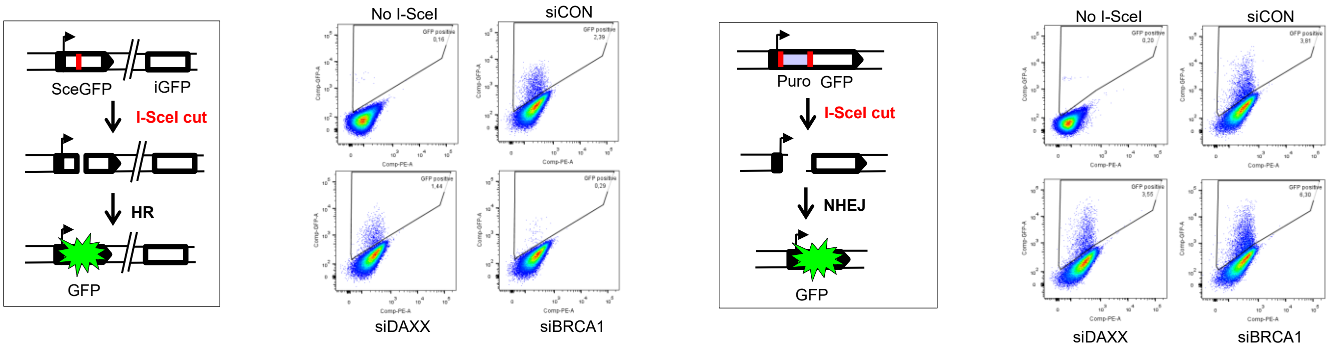

C

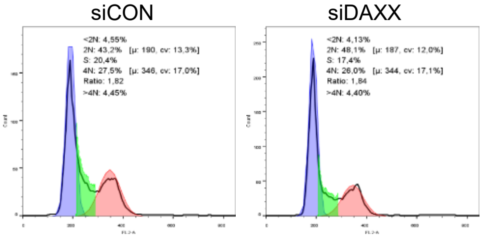

D

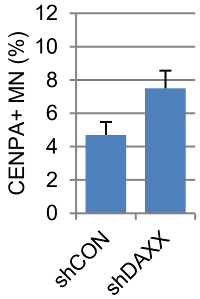

Figure S4

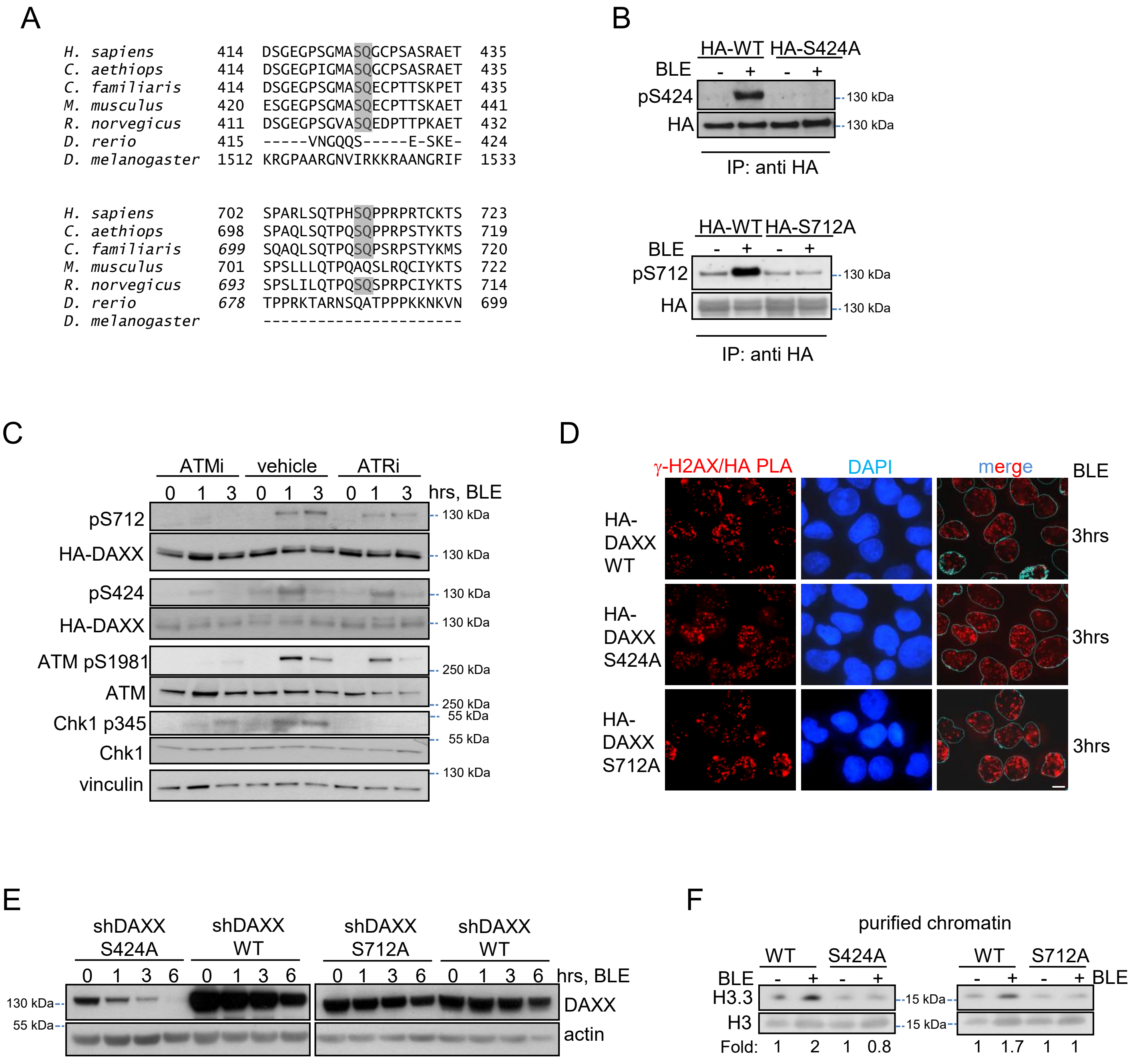
A
H. sapiens 414 DSGEGPSGMASQGCPSASRAET 435
C. aethiops 414 DSGEGPIGMASQGCPSASRAET 435
C. familiaris 414 DSGEGPSGMASQECPTTSKPET 435
M. musculus 420 ESGEGPSGMASQECPTTSKAET 441
R. norvegicus 411 DSGEGPSGVASQEDPTTPKAET 432
D. rerio 415 -----VNGQQS-----E-SKE- 424
D. melanogaster 1512 KRGPAARGNVIRKKRAANGRIF 1533
H. sapiens 702 SPARLSQTPHSQPPRPRTCKTS 723
C. aethiops 698 SPAQLSQTPQSQPPRPSTYKTS 719
C. familiaris 699 SQAQLSQTPQSQPSRPSTYKMS 720
M. musculus 701 SPSLLLQTPQAQSLRQCIYKTS 722
R. norvegicus 693 SPSLILQTPQSQSPRPCIYKTS 714
D. rerio 678 TPPRKTARNSQATPPPKKNKVN 699
D. melanogaster ----------------------
B
HA-WT HA-S424A
BLE - + - +
pS424
HA
130 kDa
IP: anti HA
HA-WT HA-S712A
pS712
IP: anti HA
C
ATMi vehicle ATRi
0 1 3 0 1 3 0 1 3 hrs, BLE
pS712
HA-DAXX
pS424
ATM pS1981
ATM
Chk1 p345
Chk1
vinculin
250 kDa
55 kDa
D
γ-H2AX/HA PLA
DAPI
merge
BLE
HA-DAXX WT
HA-DAXX S424A
HA-DAXX S712A
3hrs
E
shDAXX S424A
shDAXX WT
shDAXX S712A
0 1 3 6
hrs, BLE
DAXX
actin
F
purified chromatin
WT S424A
WT S712A
H3.3
H3
15 kDa
Fold: 1 2 1 0.8
1 1.7 1 1

Figure S5

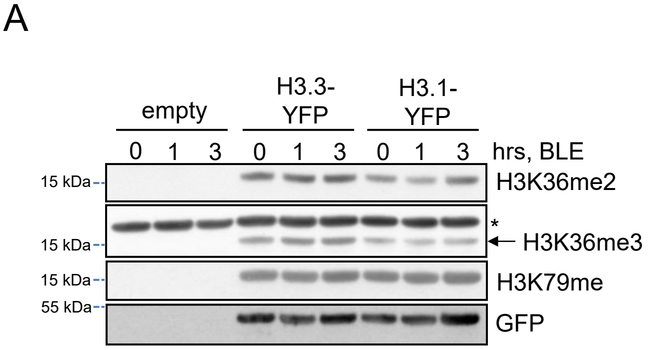
A
empty
H3.3-YFP
H3.1-YFP
0 1 3 0 1 3 0 1 3 hrs, BLE
15 kDa
H3K36me2
*
15 kDa
H3K36me3
15 kDa
H3K79me
55 kDa
GFP

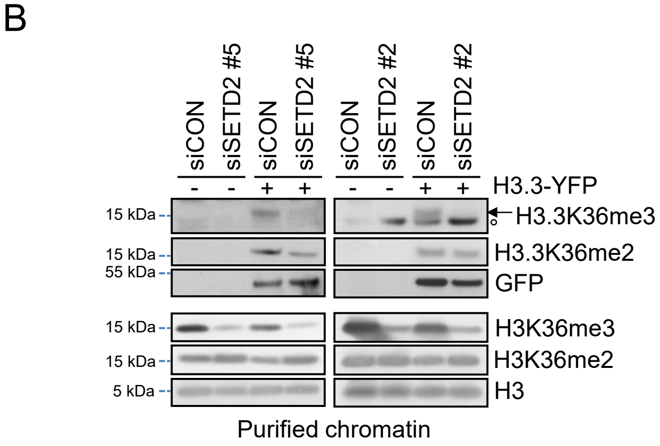
B
siCON
siSETD2 #5
siCON
siSETD2 #5
siCON
siSETD2 #2
siCON
siSETD2 #2
- - + + - - + + H3.3-YFP
15 kDa
H3.3K36me3
15 kDa
H3.3K36me2
55 kDa
GFP
15 kDa
H3K36me3
15 kDa
H3K36me2
5 kDa
H3
Purified chromatin
