# Supplementary material for: Daxx-Dependent H3.3 Deposition Promotes Double-Strand Breaks Repair by Homologous Recombination
Source: Cells. 2026 Jan 16;15(2):162. doi: 10.3390/cells15020162 (PMC12839749; doi:10.3390/cells15020162)
Supplement: Supplementary file 1 [file cells-15-00162-s001.zip › Suppl Figure Legends.pdf]

SUPPLEMENTARY FIGURE LEGENDS

Supplementary Figure S1. DAXX/H3.3 axis is regulated by DSBs induction. (A) Purified chromatin samples from MRC5, HEK293T and U2OS cells collected at the indicated times after BLE addition were assayed by immunoblot. H3.3 signals were normalized against H3 and the relative quantification of band intensities is shown as fold change, considering as 1 the untreated sample. The graph reports the means and s.d. of three independent experiments. **(B)** Stable DAXX silencing (shDAXX) was tested by immunoblotting (left) using actin as loading control and by immunofluorescence (right) with cell nuclei stained with DAPI. Scale bar represents 10μm. **(C)** Western blot performed on total lysates from U2OS cells transiently silenced with two different HIRA siRNAs (siHIRA #1 and #2). Actin represents the loading control. **(D)** Purified chromatin samples obtained from siHIRAs and mock U2OS tested for H3.3 and total H3 at the indicated times after BLE addition. Relative quantification of band intensities is shown and obtained as in Figure 1A. **(E)** Western blot performed on total lysates from U2OS cells with DAXX silencing (shDAXX). Total H3.3 was evaluated, GAPDH and Ponceau staining (histones region shown) were used as loading control. **(F)** Immunofluorescence analyses of HA-DAXX expression and localization before and after bleomycin treatment. HA-DAXX and PML bodies were revealed, respectively with an anti-HA and anti-PML antibody. Cell nuclei were stained with DAPI (blue). Scale bar represents 10μm. **(G)** Cells were exposed for 3hrs to 12μM BLE before fixation. PLA was conducted in parallel with the samples shown in Figure 1F, with antibodies against γ-H2AX and pospho-Ser15 p53. Cell nuclei were stained with DAPI (blue). Scale bar represents 10μm.

Supplementary Figure S2. DAXX-mediated deposition of H3.3 influences the apical events of the DNA damage response to DSBs. (A) U2OS cells were exposed to 12μM BLE and fixed at the indicated time points. Cells were stained by immunofluorescence with the indicated antibodies. Cell nuclei were stained with DAPI. Scale bar represents 10μm. γ-H2AX and 53BP1 foci were enumerated and plotted in the graphs (left and middle charts). The bar marks cells with less than 5 foci. Cells with less than 5 foci were considered negative (right chart). **(B)** Representative images of U2OS cells transfected with an empty vector or with H3.3-YFP or HA-DAXX^{WT}and immunostained with γ-H2AX antibody (in combination with HA antibody in the case of HA-DAXX) 1hr after 12μM BLE addition. Scale bar represents 10μm. **(C)** Cells transfected, treated and stained as in **(B)** were used to evaluate γ-H2AX foci. Cells positive for transfection and with less than 5 foci (>95% cells in untreated sample) were considered as negative. Data from three independent experiments were collected in the graph (mean and s.d.). At least 100 cells were scored for each experiment. Mock, DAXX and H3.3 samples were not statistically different using a Student's t-test analysis. **(D)**

Examples of 53BP1 staining on cell fixed before and 1hr after 12μM BLE. Cell nuclei were stained with DAPI. Scale bar represents 10μm. **(E)** Example of 53BP1 staining (red) performed on HA-DAXX (green) expressing cells fixed 3hrs after 12μM BLE. Arrows indicate cells positive for HA-DAXX expression and with less than 5 53BP1 foci. Scale bar represents 10μm. **(F)** Cells transfected with the indicated constructs were exposed to 12μM BLE and fixed after 1 or 3hrs. Immunostaining of these cells with 53BP1 antibody (in combination with FLAG antibody in the case of FLAG-H3.3) allowed to count cells positive for transfection and with less than 5 foci (negative cells). Data from three independent experiments were collected in the graph (mean and s.d.). **(G)** HEK293T cells transfected with the indicated constructs (HA-DAXX and H3.3-YFP) were exposed to 12μM BLE for 3hrs, successively fixed and stained for 53BP1 (and HA for HA-DAXX) to determine foci number (left chart). Cells positive for transfection and with less than 5 foci were considered negative (right chart). Data from three independent experiments were collected in the graph (mean and s.d.). At least 100 cells were scored for each experiment. **p<0.001 statistical significance obtained with a Student's t-test. **(H)** Same as (E) for DivA cells transiently transfected with DAXX and H3.3 encoding vectors. 4OHT was added to promote AsiSI translocation into the nucleus 4hrs before fixation to induce DSBs. **(I)** Stable shCON or shDAXX U2OS clones were treated for the indicated times with 12μM BLE. Total amount of DAXX protein (endogenous or exogenous) was evaluated by WB. Actin was used as a loading control. **(J)** U2OS cells silenced for HIRA or DAXX were concurrently transfected with H3.3-YFP or an empty vector and tested by immunofluorescence for 53BP1 foci formation 1 and 3hrs after 12μM BLE addition. Cells with less than 5 foci were considered negative. Data from three independent experiments were collected in the graph (mean and s.d.). **p<0.001 statistical significance obtained with a Student's t-test.

Supplementary Figure S3. DAXX participates in regulating DSBs repair activities. (A) Representative images of U2OS cells (stable clones of mock or shDAXX expressing a doxycicline-inducible WT DAXX protein) exposed for 24hrs to doxycicline and successively treated for 2hrs with 12μM BLE, fixed and co-stained with anti-RAD51 and -cyclin B1 antibodies. Cells with an intense cytoplasmic staining of cyclin B1 are in G2 phase of the cell cycle. RAD51 foci detectable inside cyclin B1 positive cells were enumerated (see Figure 3A). Scale bar represents 10μm. **(B)** Schematic representation of the DR-GFP (left) and EJ5-GFP (right) reporters used to monitor, respectively, homologous recombination (HR) and non-homologous end joining (NHEJ) (see Figure 3D). DR-GFP-U2OS cells and EJ5-GFP-U2OS cells silenced for DAXX (siDAXX), BRCA1 (siBRCA1) or control (siCON) were transfected with pCBASceI or empty vector (no I-SceI). After 72hrs, samples were analysed for GFP-positive cells by flow cytometry. **(C)** siCON and siDAXX cells were tested for cell cycle distribution by cytofluorimetric analysis. **(D)** shCON and shDAXX U2OS cells were

immunostained for the centromeric protein CENPA. The presence of CENPA in the MN was evaluated in 100 MN for each of three independent experiments. Data were collected in the graph (mean and s.d.) and no significant differences were revealed with a Student's t-test.

Supplementary Figure S4. DAXX is phosphorylated by ATM and ATR after genotoxic treatment. (A) The alignment of DAXX regions spanning S424 and S712 among the indicated organisms is shown. ATM/ATR consensuses (SQ) at S424 and S712 are highlighted in grey (other SQ/TQ consensus are present in the same region). **(B)** U2OS cells transfected with HA-DAXX (WT, S424A or S712A) were treated with 120μM BLE and starved 1hr later. Immunoblot analysis of total lysates were performed with the indicated antibodies. **(C)** U2OS cells transfected with HA-DAXX were pre-treated with 10μM of the ATM inhibitor KU55933 (ATMi), 2μM of the ATR inhibitor VE-822 (ATRi), or DMSO (vehicle). One hour later 120μM BLE was added and after 3hrs cells lysed. Immunoblot analysis of the total lysates obtained were performed with the indicated antibodies. **(D)** Cells expressing HA-DAXX^{WT} or phosphomutants were exposed to 12μM BLE and fixed 3hrs after BLE addition. DAXX interaction with DSBs marker γ-H2AX detected was detected by *in situ* PLA. The interactions were visualized as red fluorescent spots. Nuclei were stained with DAPI (blue). Scale bar: 10μm. **(E)** U2OS shDAXX cell lines expressing WT and phosphomutant forms of DAXX under the control of a doxycyclin-inducible promoter were tested for DAXX expression. Cells were exposed to 1μg/ml of doxycicline 24hrs before the exposure to 120μM BLE and successively harvested at the indicated time points. Actin was used as loading control. **(F)** U2OS expressing WT or mutant forms of DAXX were collected at 3hrs after 120μM BLE addition. Chromatin was purified and assayed by immunoblot. H3.3 signals were normalized against those of H3 and the relative quantification of band intensities is shown as fold change, considering as 1 the untreated sample. This experiment is one of the three independent replicates included in Figure 4A graph.

Supplementary Figure S5. H3.3 peculiar PTMs influence 53BP1 accumulation at DSBs. (A) Post translational modifications of histone H3.1-YFP and H3.3-YFP were tested on chromatin extracted from HEK293T cells. Anti GFP antibody was used to reveal the expression levels of H3.3-YFP and H3.1-YFP. Cells were treated for the indicated time with BLE 120μM. *non specific band. **(B)** U2OS cells were co-transfected with SETD2 (#2 or #5) or control (siCON) siRNAs in combination with a vector expressing H3.3-YFP (+) or a YFP (-) protein. Chromatin from these cells was tested by immunoblot with the indicated antibodies.
