# Supplementary material for: Daxx-Dependent H3.3 Deposition Promotes Double-Strand Breaks Repair by Homologous Recombination
Source: Cells. 2026 Jan 16;15(2):162. doi: 10.3390/cells15020162 (PMC12839749; doi:10.3390/cells15020162)
Supplement: Supplementary file 1 [file cells-15-00162-s001.zip › Suppl Tables_S1 and S2.pdf]

SUPPLEMENTAL MATERIAL:

Supplemental Table S1: Primary antibodies used for western blot (WB) and immunofluorescence (IF)

Antibody	Clone or Cat. #	Company	WB dilution	IF dilution
Actin	AC-74	Sigma	1:20000	
γ-H2AX	JBW301	Upstate	1:1000	1:500
H2AX	07-627	Upstate	1:2000	
53BP1	NB100-304	Novus	1:2000	1:900
DAXX	M-112	Santa Cruz	1:1000	1:50
DAXX	DAXX-03	Acris	1:1000	1:100
HA	12CA5	Roche	1:2000	1:200
HA	H6908	Sigma		1:100
DAXX pS712	This paper		1:100	
DAXX pS424	This paper		1:100	
Chk2 pT68	2661	Cell Signaling	1:1000	
Chk2	DCS-273	Enzo Life Sciences	1:800	
p53	DO1	Sigma	1:800	
Rad51	PC130	Calbiochem	1:2000	1:500
FLAG	M2	Sigma	1:1000	1:700
PML	PG-M3	Santa Cruz		1:200
Cyclin B1	GNS-1	BD-Pharmingen		1:200
H3.3	SP2	CosmoBio	1:10000	
H3.3B	GTX115549	Genetex	1:1000	
H3	GTX27843	Genetex	1:3000	
Chk1 pS345	133D3	Cell Signaling	1:1000	
Chk1	2345	Cell Signaling	1:1000	
Vinculin	hVIN1	Sigma	1:50000	
H3K4Me3	Ab8580	Abcam	1:5000	
H3K36Me2	C75H12	Cell Signaling	1:1000	
H3K36Me3	61101	ActiveMotif	1:3000	
H3K79Me1	39921	ActiveMotif	1:1000	
H3K9Me3	Ab8898	Abcam	1:2000	
H3K9Ac	C5B11	Cell Signaling	1:1000	
H3K56Ac	39082	ActiveMotif	1:2000	
GFP	3E6	Invitrogen	1:1000	
HAUSP	A300-033A	Bethyl	1:4000	
HIRA	WC119.2H11	ActiveMotif	1:1000	
ATRX	Sc15408	Santa Cruz	1:200	
Brca1	D-9	Santa Cruz	1:100	
Cyclin A	C-19	Santa Cruz	1:100	
Phospho-p53(Ser15)	9284	Cell Signaling	1:200	
ORC2	46175	Novus Biologicals	1:200	
GAPDH	SAB1405848	Merck	1:1000	

Supplemental Table S2: sh and siRNA sequences used with references.

**siRNA**	**Sequence (5'-3')**	**Ref. or Company**
siDAXX #1	GGAAGUUGGAUCUCUCAGAA	Chen and Chen, 2003
siDAXX #2	GCUCUAUGUCUACAUCAAUG	Ambion
shDAXX	GGAAGTTGGATCTCTCAGAA	This paper
Silent mutations for resistance to shDAXX	GGAAGTTGGA**C**CT**T**TCAGAA	This paper
siATRX	CAAUGGUAUUGCUACAUUU	Ambion
siHIRA #1	GGAGAUGACAAACUGAUUA	Adam et al., 2013
siHIRA #2	GAAGGACUCUCGUCUCAUG	Adam et al., 2013
siSETD2 #2	GCUCAGAGUUAACGUUUGA	Kanu et al., 2015
siSETD2 #5	UAAAGGAGGUAUAUCGAAU	Pfister et al., 2014
siBrca1	CAGCUACCCUUCCAUCAUA	Hatchi et al., 2015
siCON and shCON	CUUACGCUGAGUACUUCGA	

SUPPLEMENTAL REFERENCES:

Chen L.Y.; Chen J.D. Daxx silencing sensitizes cells to multiple apoptotic pathways. *Mol Cell Biol.* **2003,** *23,* 7108–7121. https://doi.org/10.1128/MCB.23.20.7108-7121.2003.

Adam, S.; Polo, S.E.; Almouzni, G. Transcription Recovery after DNA Damage Requires Chromatin Priming by the H3.3 Histone Chaperone HIRA. *Cell* **2013**, *155*, 94–106. https://doi.org/10.1016/j.cell.2013.08.029.

Kanu, N.; Grönroos, E.; Martinez, P.; Burrell, R.A.; Yi Goh, X.; Bartkova, J.; Maya-Mendoza, A.; Mistrík, M.; Rowan, A.J.; Patel, H.; et al. SETD2 Loss-of-Function Promotes Renal Cancer Branched Evolution through Replication Stress and Impaired DNA Repair. *Oncogene* **2015**, *34*, 5699–5708. https://doi.org/10.1038/onc.2015.24.

Pfister, S.X.; Ahrabi, S.; Zalmas, L.-P.; Sarkar, S.; Aymard, F.; Bachrati, C.Z.; Helleday, T.; Legube, G.; La Thangue, N.B.; Porter, A.C.G.; et al. SETD2-Dependent Histone H3K36 Trimethylation Is Required for Homologous Recombination Repair and Genome Stability. *Cell Rep.* **2014**, *7*, 2006–2018. https://doi.org/10.1016/j.celrep.2014.05.026.

Hatchi, E.; Skourti-Stathaki, K.; Ventz S.; Pinello L.; Yen A.; Kamieniarz-Gdula K.; Dimitrov S.; Pathania S.; McKinney, K.M.; Eaton, M.L.; et al. BRCA1 recruitment to transcriptional pause sites is required for R-loop-driven DNA damage repair. *Mol Cell.* **2015,** *57*, 636–647. https://doi.org/10.1016/j.molcel.2015.01.011.
